# No Intercellular Regulation of the Cell Cycle among Human Cervical Carcinoma HeLa Cells Expressing Fluorescent Ubiquitination-Based Cell-Cycle Indicators in Modulated Radiation Fields

**DOI:** 10.3390/ijms222312785

**Published:** 2021-11-26

**Authors:** Hisanori Fukunaga, Kiichi Kaminaga, Eri Hirose, Ritsuko Watanabe, Noriko Usami, Kevin M. Prise, Akinari Yokoya

**Affiliations:** 1 Center for Environmental and Health Sciences, Hokkaido University, N12 W7 Kita-ku, Sapporo 060-0812, Japan; 2 Institute for Quantum Life Science, National Institutes for Quantum Science and Technology, 2-4 Shirakata, Tokai 319-1106, Japan; kaminaga.kiichi@qst.go.jp (K.K.); watanabe.ritsuko@qst.go.jp (R.W.); 3 Graduate School of Science and Engineering, Ibaraki University, 2-1-1 Bunkyo, Mito 310-8512, Japan; hirose.eri@qst.go.jp; 4 Photon Factory, Institute of Materials Structure Science, High Energy Accelerator Research Organization (KEK), 1-1 Oho, Tsukuba 305-0801, Japan; noriko.usami@kek.jp; 5 Patrick G Johnston Centre for Cancer Research, Queen’s University Belfast, 97 Lisburn Road, Belfast BT9 7AE, UK; k.prise@qub.ac.uk

**Keywords:** cell cycle, fluorescent ubiquitination-based cell-cycle indicator, HeLa cell, non-targeted effect, microbeam, radiotherapy

## Abstract

The non-targeted effects of radiation have been known to induce significant alternations in cell survival. Although the effects might govern the progression of tumor sites following advanced radiotherapy, the impacts on the intercellular control of the cell cycle following radiation exposure with a modified field, remain to be determined. Recently, a fluorescent ubiquitination-based cell-cycle indicator (FUCCI), which can visualize the cell-cycle phases with fluorescence microscopy in real time, was developed for biological cell research. In this study, we investigated the non-targeted effects on the regulation of the cell cycle of human cervical carcinoma (HeLa) cells with imperfect p53 function that express the FUCCI (HeLa–FUCCI cells). The possible effects on the cell-cycle phases via soluble factors were analyzed following exposure to different field configurations, which were delivered using a 150 kVp X-ray irradiator. In addition, using synchrotron-generated, 5.35 keV monochromatic X-ray microbeams, high-precision 200 μm-slit microbeam irradiation was performed to investigate the possible impacts on the cell-cycle phases via cell–cell contacts. Collectively, we could not detect the intercellular regulation of the cell cycle in HeLa–FUCCI cells, which suggested that the unregulated cell growth was a malignant tumor. Our findings indicated that there was no significant intercellular control system of the cell cycle in malignant tumors during or after radiotherapy, highlighting the differences between normal tissue and tumor characteristics.

## 1. Introduction

Recent advances in radiotherapeutic technology have made it possible to efficiently focus the dose to the planned target volume (PTV); however, the approach to completely irradiate only tumor cells has not yet been established. Therefore, there is a spatially non-uniform dose distribution in the normal tissues surrounding the PTV, and as a result, adverse events due to radiation damage can still occur. In addition, where irradiated areas and non-irradiated areas coexist, it is known that cells that do not directly receive radiation doses receive signals from neighboring irradiated cells and behave as though they have been exposed to modified radiation fields. These well-documented responses are collectively known as the non-targeted radiation effects, although the underlying molecular mechanisms remain to be determined [1]. Several studies have shown significant alterations in cell survival in vitro in spatially modified radiation fields, experimentally [2,3,4] and theoretically [5,6,7]. This impact on cell survival is partially driven by intercellular signaling via gap junctions or soluble factors between irradiated cells and non-irradiated cells in living systems [8,9,10,11]. However, the non-uniform, radiation-induced impacts on the cell cycle have not been fully determined, although the cell cycle is closely related to cell death. From the point of view radiation biology, and also clinical oncology, it is important to understand how the cell cycle is regulated in spatially modulated radiation fields.

A fluorescent ubiquitination-based cell-cycle indicator (FUCCI) technique has been developed to easily and clearly visualize the cell-cycle phases using fluorescence microscopy [12,13]. While Cdt1 is a deoxyribonucleic acid (DNA) replication-licensing factor expressed in the G1 phase, the expression of the Cdt1 inhibitor, Geminin, was observed in the S/G2/M phases. Thus, the protein levels of Cdt1 and Geminin oscillated inversely [12]. The cells that expressed FUCCI changed from red in the G1 phase to yellow in the G1/S interphase and then to green in the S, G2, and M phases in the fluorescence microscope field. Geminin and Cdt1, which fused to a green fluorescent reporter and red fluorescent reporter, respectively, were expressed at specific points in the cell cycle. Therefore, this technique is a useful approach for tracking cell-cycle progressions in individual cells using live-cell fluorescence imaging. In fact, previous studies have shown that cell-cycle arrest can be visualized using a treatment of agents, such as drugs [14,15,16,17,18] and ultraviolet (UV) irradiation [19].

**Figure 1:**
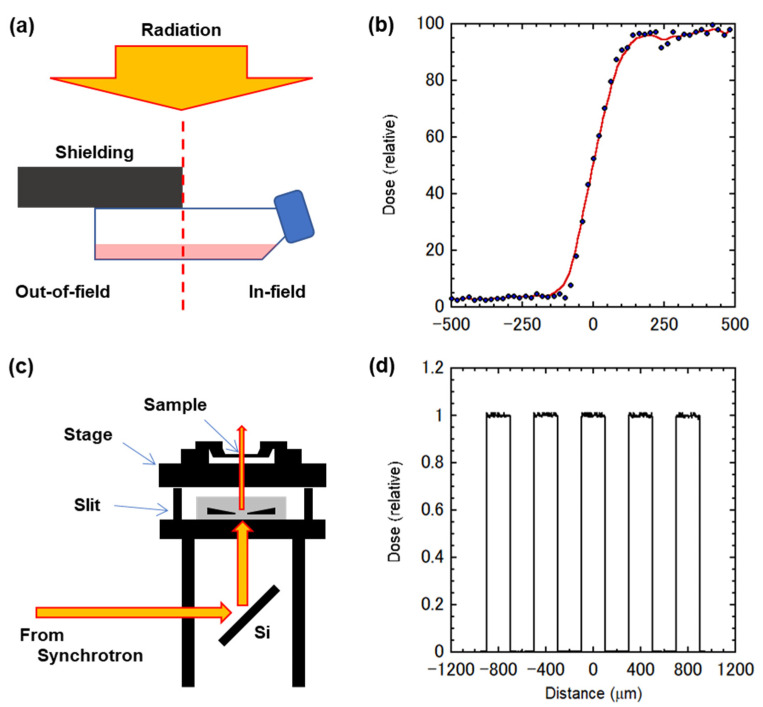
Irradiation settings and the dose profiles. (**a**) Schematic representation of the half irradiation field. Cells were irradiated at 8 Gy in a single T25 flask. (**b**) Dose profiles of the half irradiation field, calculated with PHITS code. A 0.5 cm exclusion zone, where cells were not analyzed, was established to allow for uncertainties in the setup and avoid analysis at any steep dose gradients. (**c**) Schematic representation of the 200 μm-wide microbeam irradiation setting. (**d**) Dose profiles of the 200 μm-wide microbeams, calculated with PHITS code. The beam intensity was essentially flat within the beam width.

In the current study, we hypothesized that the non-targeted effect on the regulation of the cell cycle can induce significant alterations in cell survival in modified radiation fields. To approach the hypothesis, we employed a subline of the human cervical carcinoma HeLa cells that expresses the FUCCI (HeLa–FUCCI cells). The non-targeted effect on the cell cycle was analyzed after their exposure to a modulated radiation field configuration, where half of the cell population was shielded, that delivered non-uniform dose distributions. The non-uniform dose profiles for each configuration are shown in Figure 1a,b. Thus, there were four sample groups, namely the uniform, in-field, out-of-field, and no (control) exposures. The dose distributions were confirmed using Gafchromic XR-RV3 radiochromic film (Ashland Inc., Covington, KY, USA), which showed that the scattered dose under the shielding was approximately 2–3% of the full dose delivered to the exposed region [2]. This setting was considered to be suitable for the detection of non-targeted effects via soluble factors. In addition, using synchrotron X-ray microbeams [20], high-precision 200 μm-slit microbeam irradiation was performed. As shown in Figure 1c,d, in this study, we employed high-precision 200 μm-slit irradiation (using center-to-center distances of 400 μm). The microbeam size was adjusted using a four-dimensional slit system. As previously described [21], we applied a Monte Carlo particle transport simulation code, PHITS ver. 2.96 [22], to calculate the micro-slit dose profiles. Doses delivered by the secondary electrons to the outside (valley) region of the microbeam area were negligible (0.25%) due to their short range (1.1 μm maximum). This was considered suitable for the detection of non-targeted effects via gap junctions or cell–cell contacts.

We previously demonstrated that the cell-cycle phases of irradiated HeLa cells can be modulated following exposure to conventional 150 kVp X-rays [23] and synchrotron-generated 5.35 keV monochromatic X-rays [24]. In addition, we reported the fates of both irradiated cells and non-irradiated bystander cells in the HeLa–FUCCI cell population using time-lapse imaging [25]. The result suggested that the cell-cycle distributions of the bystander cells were not significantly affected by microbeam exposure of the selected cells in a small colony composed of 20 cells or less, although some bystander cells showed cell death after a few cell divisions. However, the non-targeted, radiation-induced impacts on the cell-cycle regulation, focusing on spatially non-uniform dose distribution, remain to be determined. Further investigations with the FUCCI method will be imperative to offer new insights into the non-targeted radiation effects and their clinical applications for cancer treatment.

## 2. Results and Discussion

**Figure 2:**
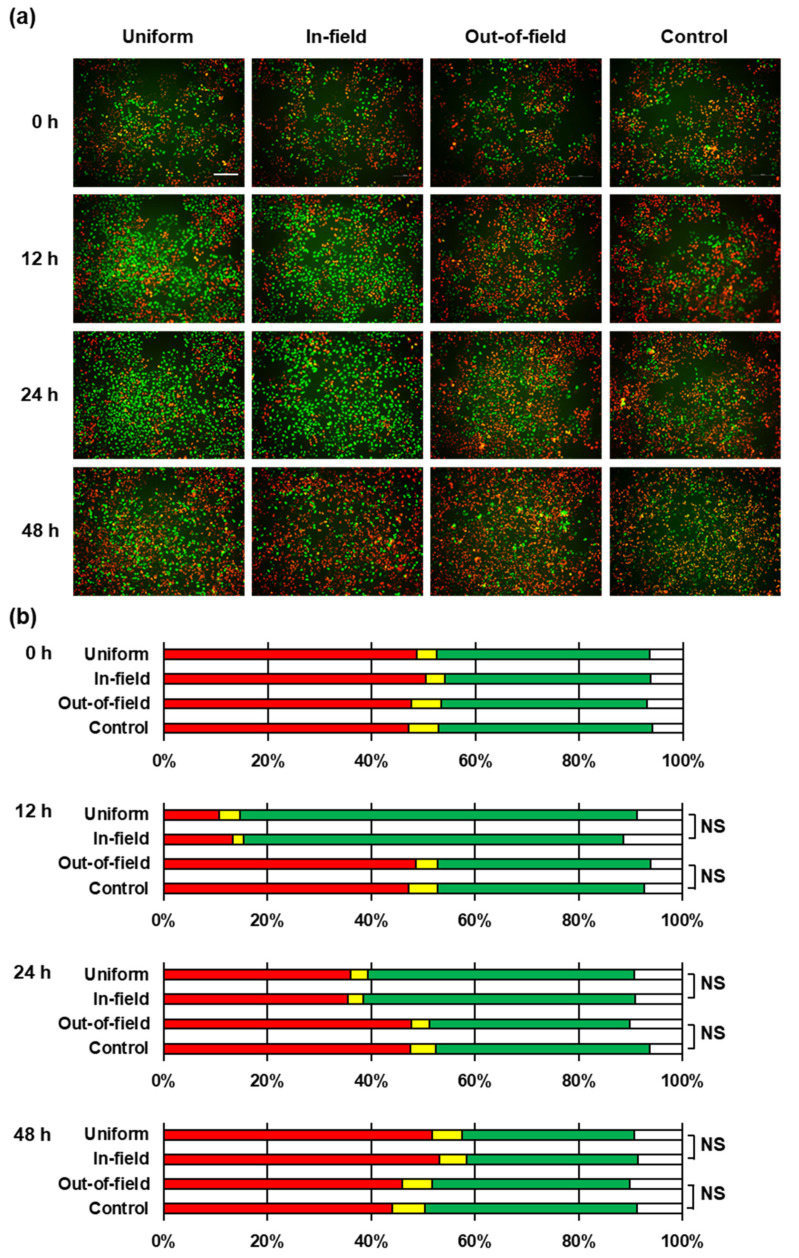
Cell-cycle distributions of HeLa–FUCCI cells following exposure to modulated radiation fields. (**a**) Representative fluorescent images of HeLa–FUCCI cells 0, 12, 24, and 48 h following exposure to the half irradiation field (refer to Figure 1). Cell nuclei are indicated by the red, yellow, green, and colorless regions depending on their cell cycles: G1, G1/S, S/G2, and M, respectively. Scale bar, 100 μm. (**b**) Summary graph of fluorescent color distributions in HeLa–FUCCI cells after the control, out-of-field, in-field, and uniform exposures. The color of the bars indicates the color of the cells, respectively. There was no difference in the cell-cycle distribution between the uniform and the in-field exposures and between the control and the out-of-field exposures, which suggested that there was no detectable, non-targeted effect. NS stands for *Non-Significant* (chi-square test).

We confirmed the cell-cycle arrest in the G2/M phase following exposure to 8 Gy X-rays. As shown in Figure 2a, up to approximately 24 h after irradiation, release from the arrest was clearly observed, and then redistribution to the control state gradually occurred. This result supported the reproducibility of previous reports [19,20]. HeLa–FUCCI cells were able to bypass the G1 checkpoint, whereas the G2/M checkpoint was functional. HeLa–FUCCI cells were probably functionally p53 deficient; however, this detail remains to be determined. HeLa cells that expressed wild-type TP53 and had no mutations were, nonetheless, identified as P53 null when the E6 protein expressed from an endogenous papillomavirus degraded TP53 and left no protein to be detected using Western blot analysis [26]. Thus, the G1/S checkpoint governed by p53 activated through ATM and its downstream factor, Chk2, did not function properly in HeLa cells [27]. The G2/M checkpoint, by contrast, worked via a pathway through which the cell-cycle check point factors, Chk1 and Cdc25, were activated by ATR [28]. The G2/M cell-cycle arrest became remarkable, which indicated that the release from radiation-induced, cell-cycle arrest required cellular processes, which presumably sustaind the Chk1’s inhibition pathway of Cdc2/Cyclin B phosphorylation by Cdc 25 [29].

Figure 2b shows the temporal changes in the fluorescent color distributions in HeLa–FUCCI cells after the uniform, in-field, and out-of-field exposures. The fluorescent colors of HeLa–FUCCI cells showed their cell-cycle progressions, suggesting radiation-induced impacts. The green HeLa–FUCCI cells accounted for more than 80% of the total cells 12 h after the uniform and in-field exposures. However, the chi-square test showed that there was no difference in the cell-cycle distribution between the control exposure and the out-of-field exposure, and between the in-field exposure and the uniform exposure. According to several previous studies [8,9,10,11], the non-targeted effects can induce significant alternations in the cell survival via soluble factors following exposure to modified radiation fields. However, in this study, the impact on the cell cycle could not be detected.

**Figure 3:**
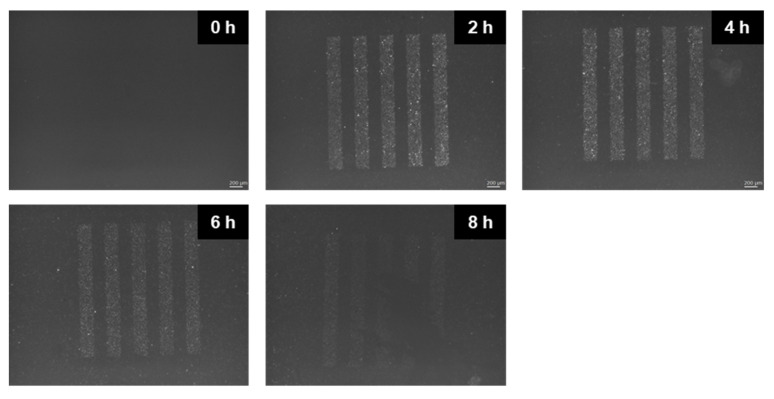
γ-H2AX-stained images of HeLa–FUCCI cells following exposure to 200 μm-slit X-ray beams. Cells were irradiated using 200 μm-slit X-ray beams. Representative γ-H2AX-stained images of FUCCI cells 0, 2, 4, 6, and 8 h after irradiation. The distribution of immune-stained γ-H2AX in the sample was a good approximation of the shape of the 200 μm-slit irradiation patterns. Scale bar, 200 μm.

Next, using microbeams, we further verified the above results. We employed the staining of γ-H2AX to confirm the 50% 200 μm-slit irradiated areas. Figure 3 shows the representative images of HeLa–FUCCI cells 0, 2, 4, 6, and 8 h after exposure to 10 Gy 200 μm-slit-modulated X-rays. The γ-H2AX foci formation confirmed DNA double-strand breaks (DSBs) in the nucleus, and the distribution in the culture clearly depended on the shape of the irradiated area (peak region).

**Figure 4:**
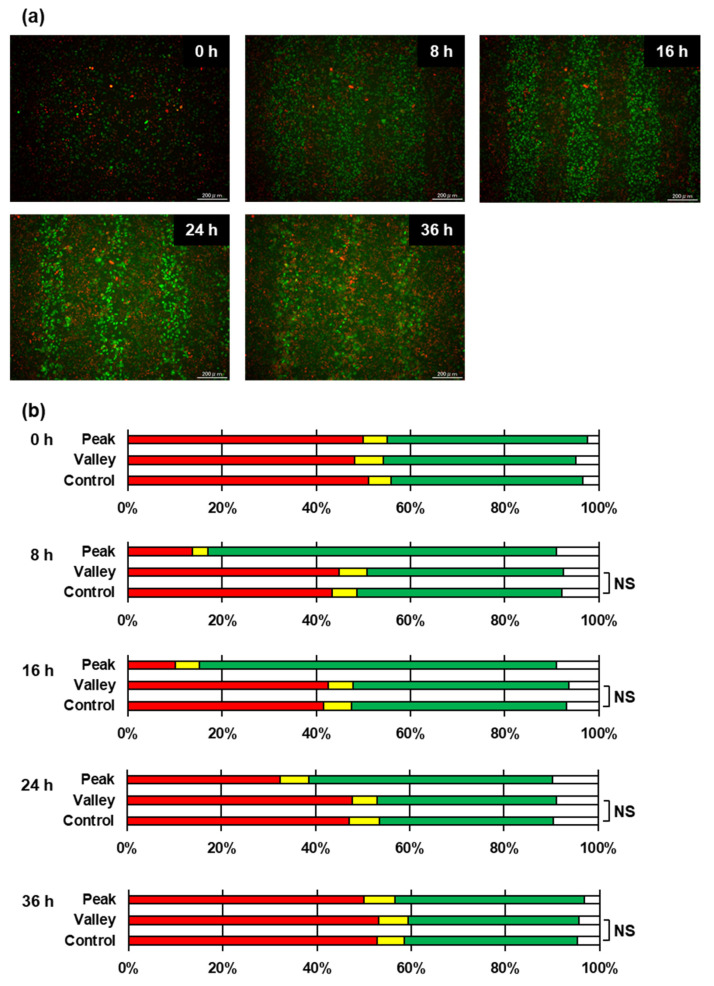
Time-lapse imaging of HeLa–FUCCI cells following exposure to 200 μm-slit X-ray beams. (**a**) Representative fluorescent images of HeLa–FUCCI cells 0, 12, 24, and 48 h following exposure to the 200 μm-wide microbeams (refer to Figure 1). Cell nuclei are indicated by the red, yellow, green, and colorless regions depending on their cell cycles: G1, G1/S, S/G2, and M, respectively. Scale bar, 200 μm. (**b**) Summary graph of fluorescent color distributions in HeLa–FUCCI cells in the control, valley, and peak regions. The color of the bars indicates the color of the cells, respectively. There was no difference in the cell-cycle distribution between the control and the valley regions, which suggests that there was no detectable non-targeted effect. NS stands for *Non-Significant* (chi-square test).

To investigate the non-targeted effects on the regulation of the cell-cycle, we applied time-lapse imaging to investigate the cell-cycle arrest and release of HeLa–FUCCI cells following exposure to 8 Gy 50% 200 μm-slit-modulated X-ray beams (Figure 4). The time variation in the cell-cycle arrest was inconsistent with the kinetics of γ-H2AX foci formation, as DSB rejoining was a faster process than the passage of the cell cycle checkpoint that progressed within several hours [30]. The chi-square test showed no difference in the cell-cycle distribution between the control (non-irradiated) and the valley regions.

In this study, using intensity-modulated radiation fields and the FUCCI technique, we investigated the non-targeted effects of radiation on the cell cycle in HeLa cells. It is known that spatial constraints (i.e., limitations on available space due to the presence of neighboring cells) impose constraints on cell functions, including cell cycles and proliferation, although it remains unclear whether mechanical constraints control cell-cycle progression in cell populations and at what stage of the cell cycle this regulation may occur [31,32]. However, in the current study, the intercellular control of the cell cycle in HeLa cells, via soluble factors and cell–cell contacts, following exposure to modulated and micro-slit X-ray fields was not detected, which suggested that the unregulated cell growth was a malignant tumor. Cancer cells that undergo uncontrolled cell-cycle progression and cell-cycle checkpoints need to be defective for a cell to become cancerous. There is a possibility that a signaling pathway in the regulation of the cell cycle, if it exists at all, does not properly work in p53 and its downstream p21 pathway-deficient cells. In addition, as suggested by the results of this study, the intercellular regulatory mechanisms for the cell cycle may not function in malignant tumors. Thus, it may be that there is no synergy between chemotherapy with cell-cycle inhibitors and non-targeted effects associated with radiation therapy.

Modern radiotherapy techniques, such as intensity-modulated radiotherapy, image-guided radiotherapy, and tomotherapy, can conform precious dose distributions to target tumors, thereby reducing the adverse effects in normal surrounding tissues. However, such heterogeneous dose distributions have been shown to cause non-targeted effects that are mediated by intercellular signaling from irradiated cells in high-dose regions to those in low-dose regions. From the point of view of radiation biology, and also clinical oncology, further investigations on the loss of intercellular control following exposure to modulated radiation fields will offer genuine promise for further understanding the characteristics of cancer cells, as well as novel radiotherapeutic approaches. As a next step, we would prefer to use flow cytometry to examine the criminal changes in the cell cycle of irradiated and non-irradiated cells, for example, in cells without FUCCI or other cancer cells.

## 3. Materials and Methods

### 3.1. Cell Culture

A subline of the HeLa–FUCCI cells, RCB2812 HeLa.S–FUCCI, which is a cell-cycle marker [12], was provided by the RIKEN BioResource Center in Japan. The cells were cultured in Dulbecco’s Modified Eagle’s medium (Wako Pure Chemicals, Osaka, Japan), which contained 10% fetal bovine serum (Biological Industries, Kibbutz Beit-Haemek, Israel) and 1% antibiotic-antimycotic (Life Technologies, Carlsbad, CA, USA), in a humidified incubator maintained at 37 °C in an atmosphere with 95% air and 5% CO_2_. The HeLa–FUCCI cell doubling time was approximately 18 h under the conditions. Pre-cultured HeLa cells were seeded into a T25 flask (Nunclon surface NUNC) or a 35 mm diameter dish (Falcon 35 mm Easy-Grip dish) in order to reach 90–100% confluency the following day. The rate was set at a relatively high level, considering the possibility of signal transduction by cell–cell contacts.

### 3.2. Conventional X-ray Settings and Modulated Fields

As previously reported [23], the cells were exposed to X-rays via the lid of a culture dish using an X-ray generator with a W-target that was operated at a tube voltage of 150 kVp (Softex, Kanagawa, Japan), which was operated at a tube voltage of 150 kV, and a tube current of 4.1 mA. The characteristic X-rays from the tungsten anode were applied to expose the samples, and the most intense energy was approximately 60 keV. The 0.2 mm aluminum filter was applied to filter the X-rays that were lower than 7 keV. The dose rate was 1 Gy/min, and the total doses of the X-rays absorbed by the cells were set to be 8 Gy because preliminary tests showed that it was difficult to detect the G2/M arrest in HeLa–FUCCI cells at doses lower than 8 Gy. We also utilized an XRAD225 X-ray cabinet (Precision X-ray Inc., Bradford, PA, USA) to confirm the reproducibility of the results (13.3 mA, 225 kVp, 2 mm Cu filtered, 0.59 Gy/min, and 8 Gy).

To achieve non-uniform dose distributions, in which half of the cell population was shielded, we applied a Pb shielding material downstream of the X-ray source. Cells were irradiated in a T25 flask, with the exclusion region omitted from the analysis. A 0.5 cm “exclusion region” was set where cells were not analyzed, which allowed for uncertainties in the setup and avoided analysis at any steep dose gradients. Doses given by strayed secondary electrons from the exposed area were also not considered.

### 3.3. Synchrotron X-ray Microbeam Settings and Micro-Slit Fields

The 5.35 keV monochromatic X-ray microbeam irradiation was performed using the synchrotron beamline BL-27 at the Photon Factory of the High Energy Accelerator Research Organization (KEK) in Japan [33].

Synchrotron-generated X-rays from the Photon Factory comprised a source of quasi-parallel X-ray microbeams that could be employed as a powerful probe to target specific sites in a living system, including tissues [34]. The ability to select individual cells or regions of tissues for localized irradiation was the key to determining the role of intra- and inter-cellular signals.

### 3.4. FUCCI Imaging

To visualize red (570 nm) fluorescence emissions and green (505 nm) fluorescence emissions from the cell nuclei, a fluorescence microscope BZ-X700 (KEYENCE, Osaka, Japan) equipped with an automatic filter wheel was employed. The cycling of fluorescent colors in HeLa–FUCCI cells was expressed as expected—red→yellow→green→colorless→red—which reflected progression via different cell-cycle stages (G1→G1/S→S/G2→M→G1).

### 3.5. Immunochemical Staining

Immunochemical staining for serine-139 phosphorylated histone H2AX (γ-H2AX) was performed to confirm the irradiated regions following exposure to 200 μm-slit-modulated X-ray beams. For immunofluorescence staining, cells were fixed with 2% paraformaldehyde in phosphate-buffered saline (PBS) and treated with 0.5% Triton X-100 (Sigma, St. Louis, MO, USA). To block and dilute primary or secondary antibodies, 5% goat serum (ThermoFisher scientific, Waltham, MA, USA) in PBS was employed. Primary mouse monoclonal anti-γ-H2AX antibody (Merck Millipore, Billerica, MA, USA) and secondary goat anti-mouse Alexa Fluor 647 (ThermoFisher scientific, Waltham, MA, USA) were employed.

### 3.6. Data Analysis

We observed more than 100 cells in each group, namely the uniform, in-field (peak), and out-of-field (valley) exposures, as well as the non-irradiated (control) from more than three independent experiments on different days. The statistical significance in the cell-cycle distributions was determined using the chi-square test. Statistical significance was defined as *p* ≤ 0.05.

## 4. Conclusions

To our knowledge, using synchrotron X-ray microbeams, high-precision 200 μm-slit irradiation was first performed to investigate non-targeted radiation effects on the intercellular regulation of the cell cycle in cancer cells. In this study, with intensity-modulated radiation fields and the FUCCI technique, we investigated the non-targeted radiation effects on the cell cycle in HeLa cells. We found that intercellular regulation of the cell cycle via soluble factors and cell–cell contacts following irradiation was hardly detected, indicating the uncontrolled cell-cycle progression.

## Data Availability

Not applicable.
